# The Epidemiology of COVID 19 in the Amazon and the Guianas: Similarities, Differences, and International Comparisons

**DOI:** 10.3389/fpubh.2021.586299

**Published:** 2021-03-11

**Authors:** Mathieu Nacher, Cyril Rousseau, Tiphanie Succo, Audrey Andrieu, Mélanie Gaillet, Céline Michaud, Véronique Servas, Maylis Douine, Roxane Schaub, Antoine Adenis, Magalie Demar, Philippe Abboud, Loïc Epelboin, Félix Djossou

**Affiliations:** ^1^Centre d'Investigation Clinique Antilles Guyane, INSERM 1424, Centre Hospitalier de Cayenne, Cayenne, French Guiana; ^2^Département Formation Recherche (DFR) Santé, Université de Guyane, Cayenne, French Guiana; ^3^Santé Publique France, Cayenne, French Guiana; ^4^Centres Délocalisés de Prévention et de Soins, Centre Hospitalier de Cayenne, Cayenne, French Guiana; ^5^Laboratory, Centre Hospitalier de Cayenne, Cayenne, French Guiana; ^6^Unité Mixte de Recherche Tropical Biome et Immuno-Pathologie (UMR TBIP), Université de Guyane, Cayenne, French Guiana; ^7^Service des Maladies Infectieuses et Tropicales, Centre Hospitalier de Cayenne, Cayenne, French Guiana

**Keywords:** COVID 19, epidemiology, testing, mortality, Amazon, Guiana shield, vulnerable populations

## Abstract

**Background:** The COVID 19 epidemic submerged many health systems in the Amazon. The objective of the present study was to focus on the epidemic curves of the COVID 19 epidemic in different centers, and to look at testing and mortality data.

**Methods:** Publicly available datasets were used. The log_10_ of the daily cumulated number of cases starting from the day the territory reached 100 cumulated cases was plotted to compare the magnitude, shape and slope of the different curves. The maximum daily testing efforts were plotted for each territory in relation to the maximum daily number of diagnoses. The case fatality rate was computed by dividing the number of COVID 19 deaths by the number of confirmed cases.

**Results:** In the Amazonian regions in general the speed of growth was generally lower than in Europe or the USA, or Southern Brazil. Whereas, countries like South Korea or New Zealand “broke” the curve relatively rapidly the log linear trajectory seemed much longer with signs of a decline in growth rate as of early July 2020. After a very slow start, French Guiana had the lowest slope when compared to other Amazonian territories with significant epidemics. The Amazonian states of Roraima, Amazonas, Parà, and Amapà had among the highest number of cases and deaths per million inhabitants in the world. French Guiana had significantly fewer deaths relative to its number of confirmed cases than other Amazonian territories. French Guiana had a late epidemic surge with intense testing scale-up often exceeding 4,000 persons tested daily per million inhabitants. Brazil was an outlier with low daily testing levels in relation to the number of daily diagnoses.

**Conclusions:** There were marked heterogeneities mortality rates suggesting that socioeconomic, political factors, and perhaps ethnic vulnerability led to striking outcome differences in this Amazonian context.

## Introduction

South America was affected by the COVID 19 epidemic after Asia, Europe, and North America, but the epidemic eventually caught up and overwhelmed the health system (1). This was perhaps best epitomized by the news images of the tragedy unfolding in Manaus, in the heart of the Amazon, as well as Guayaquil in Ecuador, or several cities in Peru (2, 3). Early in the epidemic, researchers studied the relation with climatic variables, and as for influenza (4–6), humidity and temperature were shown to have some impact on the reproductive number of COVID 19 (7–15). It has also been suggested that ultraviolet A and B radiation were associated with a reduction of the mortality of COVID 19 (16–18). Young populations, which is the case for the Amazonian region, are described as much less at risk of severe complications than older age groups (19). It was therefore somewhat unexpected to discover that Northern Brazil, with its hot and humid equatorial climate was one of the most affected regions in the world for COVID 19 (20). The Amazon basin and the Guiana shield are covered by a dense primary forest, roadways are therefore scarce and river or air travel are the main connecting routes between many villages and cities. Apart from the major cities, population density is very low, and poverty widespread notably in the favelas where the local population density is high and social distancing is difficult to implement in practice (21). The density of health professionals and hospital beds is also lower than in other parts of South America (22–24). Although, there are geographic commonalities, there are also differences in health expenditure per capita, differences in the organization of prevention testing and care, and differences in political leadership in confronting the crisis. Despite unprecedented research efforts and discoveries, the present pandemic is still incompletely understood, and its future uncertain. Describing and comparing trends and indicators between regions often yields instructive insights. In the present study, the objective was hence to focus on the dynamics of the COVID 19 epidemic in a singular region, the Amazon and the Guiana Shield, more specifically the Brazilian states of Amapa, Para, Roraima, and Amazonas, and in French Guiana and Suriname. A secondary objective was then to compare the Amazon and the Guiana shield to other regions of the world.

## Methods

### Data Sources

Data sets were downloaded from https://ourworldindata.org/coronavirus (1), which updates global data on COVID 19 (notably countries from continental Europe, North America, South America, East Asia, South East Asia…). Because the greater Amazonian area includes different countries or regions of countries, we use other data sources to obtain more detail, notably from the Brazilian ministry of health website (20), which entails state data. For French Guiana, a French overseas territory located on the Guiana shield, between Surinam an the Brazilian State of Amapá, the data was obtained from Santé Publique France (25), the French centers for disease control. The cumulated testing data was obtained from https://www.worldometers.info/coronavirus/ (26).

### Comparisons

In order to compare the epidemic growth, we plotted the log_10_ of the daily cumulated number of cases starting from the day the territory reached 100 cumulated cases. This allowed to compare the magnitude, shape and slope of the different curves. The median R_t_ values were computed for Amazonian territories.

The magnitude of the maximum daily testing efforts in relation to the maximum daily number of diagnoses were also compared. Cumulated number of tests were also plotted in relation to the total number of COVID 19 deaths.

The case fatality rate was computed by dividing the number of COVID 19 deaths by the number of confirmed cases.

### Data Analysis

Data was analyzed with STATA 15. In order to track the epidemic dynamics, we computed the effective reproduction number, which is the average number of new infections caused by a single infected individual at time *t* in the partially susceptible population. Indeed varying proportions of the population are immune to any given disease at any given time and social distancing may vary over time. The R_t_ values were calculated using the R EpiEstim package which computes R from daily new cases of COVID 19. Median values for each territory were plotted on a single graph.

### Regulatory and Ethical Considerations

The study used public anonymous aggregated data and did not require any ethical review according to French Authorities.

## Results

### Comparison of Cumulated Case Numbers

**Figure 1:**
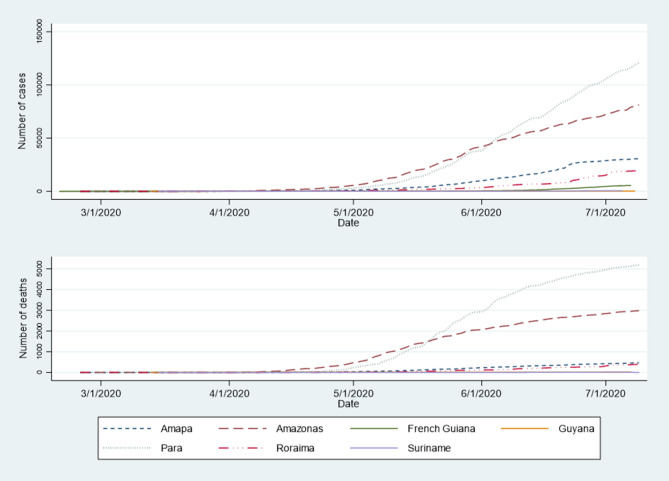
Evolution of the number of COVID-19 cases and deaths in Amazonian territories.

Figure 1 shows the chronological growth of the number of cases and deaths in Amapa, Amazonas, Para, Roraima, French Guiana, Guyana, and Suriname. At the normal scale the Guiana shield territories are hardly visible given the disproportionately higher case and death numbers in Brazil. The much larger population in Brazil relative to the Guiana shield mostly concerns Amazonas (3,874,000) and Para (8,074,000) whereas Amapa (751,000) and Roraima (496,936) were closer in scale to the population of the Guiana Shield [French Guiana (290,000), Guyana (779,004), and Suriname (575,991)], yet there were still much greater numbers of COVID 19 cases and deaths.

**Figure 2:**
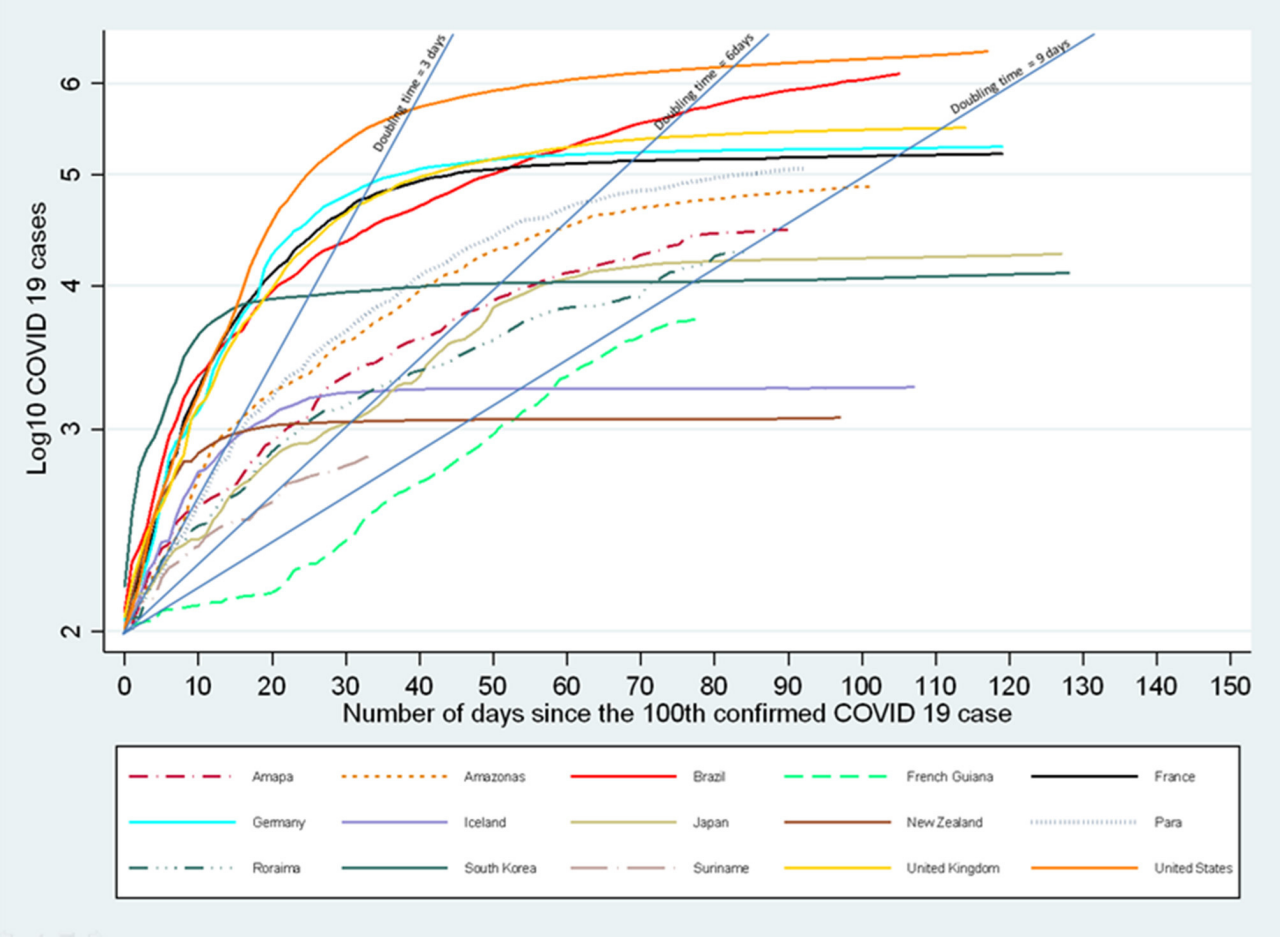
Evolution of the number of COVID-19 cases in selected countries and Amazonian territories.

Figure 2 shows the evolution of the log_10_ of the number of COVID-19 cases in selected countries. Regarding the Amazonian regions in general, the speed of growth was generally lower than in Continental Europe or the USA, or Southern Brazil. The slope was somewhat parallel to that of Japan. Whereas, countries like South Korea or New Zealand “broke” the curve relatively rapidly the loglinear trajectory seemed much longer with signs of a decline in growth rate as of early July 2020. After a very slow start, French Guiana had the lowest slope when compared to other Amazonian territories with significant epidemics (Guyana only declared 284 cases so far and was not plotted).

### Plotting Number of Deaths and Cases per Territory

**Figure 3:**
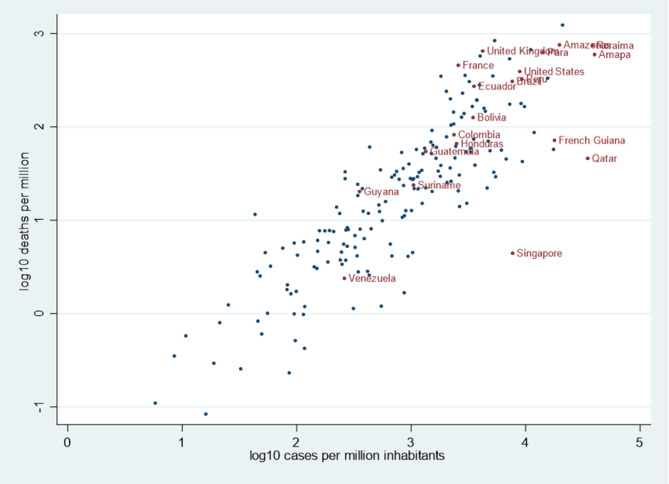
Scatterplot of the log_10_ of the number of deaths and the log_10_ of the number of cases per million inhabitants in selected countries and Amazonian territories.

Figure 3 shows a scatterplot of the log_10_ of the number of deaths per million inhabitants and the log_10_ of the number of cases per million inhabitants. This shows the loose positive correlation of the number of deaths and the number of cases, and more importantly it shows that countries on the lower right side of the scatterplot have markedly less deaths that those on the upper left part of the plot. Hence among Amazonian territories Figure 3 shows that the Amazonian states of Roraima, Amazonas, Pará, and Amapá had among the highest number of cases and deaths per million inhabitants in the world. Other states with Amazonian territories (Colombia, Ecuador, Bolivia, Venezuela) are represented with pooled country data which does not allow to disaggregate the Amazonian population segment. Guyana despite a low number of confirmed cases had significant mortality and Suriname was in an intermediary position. French Guiana had significantly fewer deaths relative to its number of confirmed cases than other Amazonian territories. Supplementary Figure 1 shows the relation between median age and case-fatality rate showing the relative youth of French Guiana and Amazonian territories.

### Proportion of Confirmed Cases That Died

Table 1 represents the detailed data on July 7th and shows that the proportion of deaths per case ranged from 0.4% In French Guiana to 7.7% in Ecuador, with among Amazonian territories Guyana with 5.7% mortality despite a low number of cases.

**Table 1:** Comparison of total number of COVID-19 cases and deaths, per million inhabitants and respective proportion of deaths/cases (July 7th 2020).

**Territory**	**Total cases**	**Total deaths**	**Cases per million population**	**Deaths per million population**	**Deaths per 100 cases**
Amapa	30,004	449	39,952	598	1.50
Amazonas	76,424	2,938	19,727	758	3.84
Bolivia	40,509	1,476	3,470	126	3.64
Colombia	120,281	4,210	2,364	83	3.50
Ecuador	62,380	4,821	3,536	273	7.73
French Guiana	5,469	22	18,858	76	0.4
Guyana	278	16	353	20	5.76
Para	114,535	5,105	14,185	632	4.46
Peru	305,703	10,772	9,272	327	3.52
Roraima	18,948	371	38,129	746	1.96
Suriname	614	14	1,047	24	2.28
Venezuela	7,411	68	261	2	0.92

### Cumulated Test Numbers and Deaths

**Figure 4:**
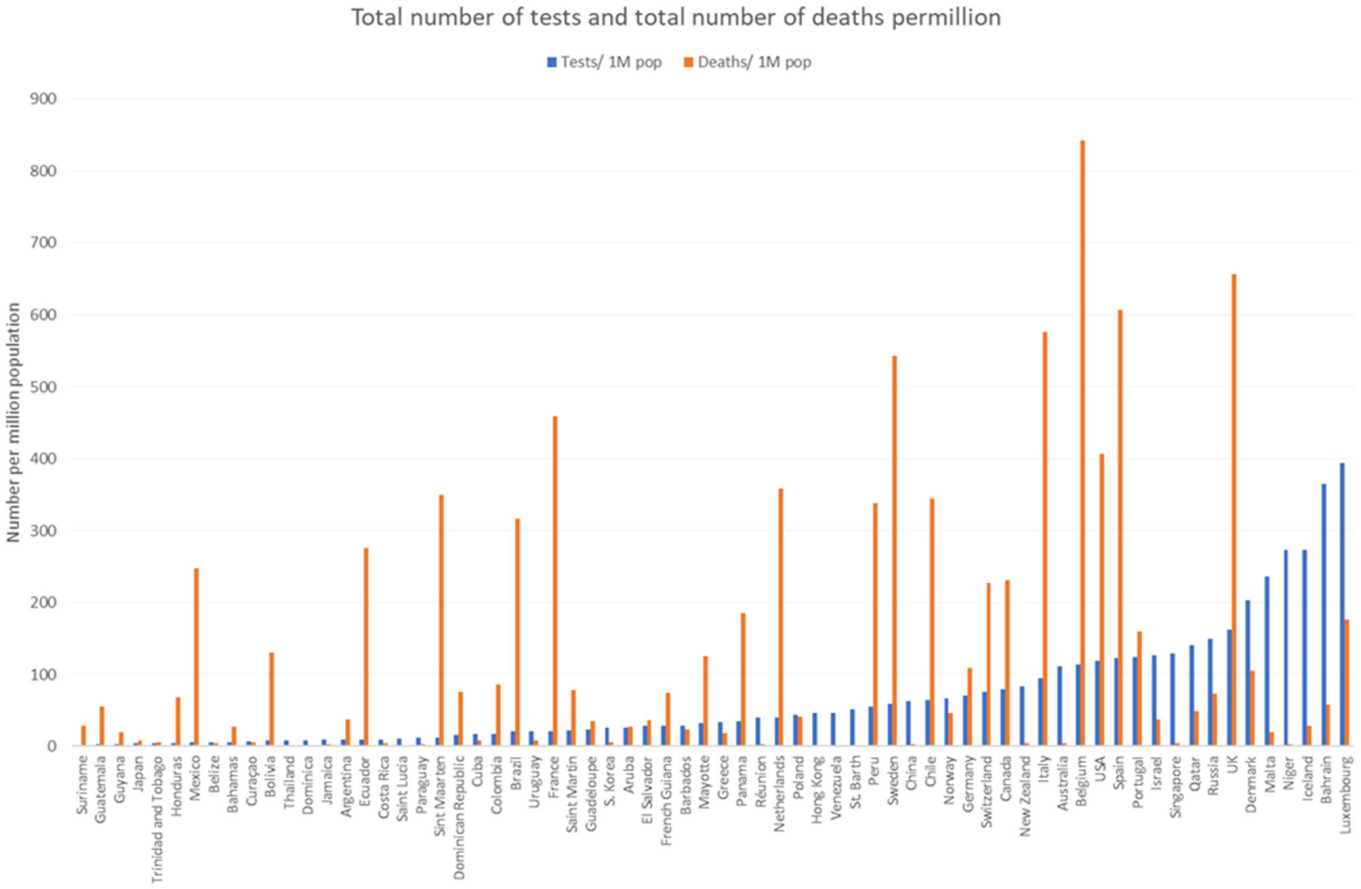
Comparison of the total number of diagnostic tests and deaths per million inhabitants.

Figure 4 does not include details on the total number of persons tested for Brazilian Amazonian states; it shows that in some countries the large number of cumulated tests was associated with a large number of deaths; in other countries the large number of tests was associated with few deaths, presumably reflecting different strategies. Brazil and continental France for example had relatively few tests but large numbers of deaths, whereas Iceland and Israel had intense testing with low mortality.

### Maximum Number of Daily Tests and Diagnoses

**Figure 5:**
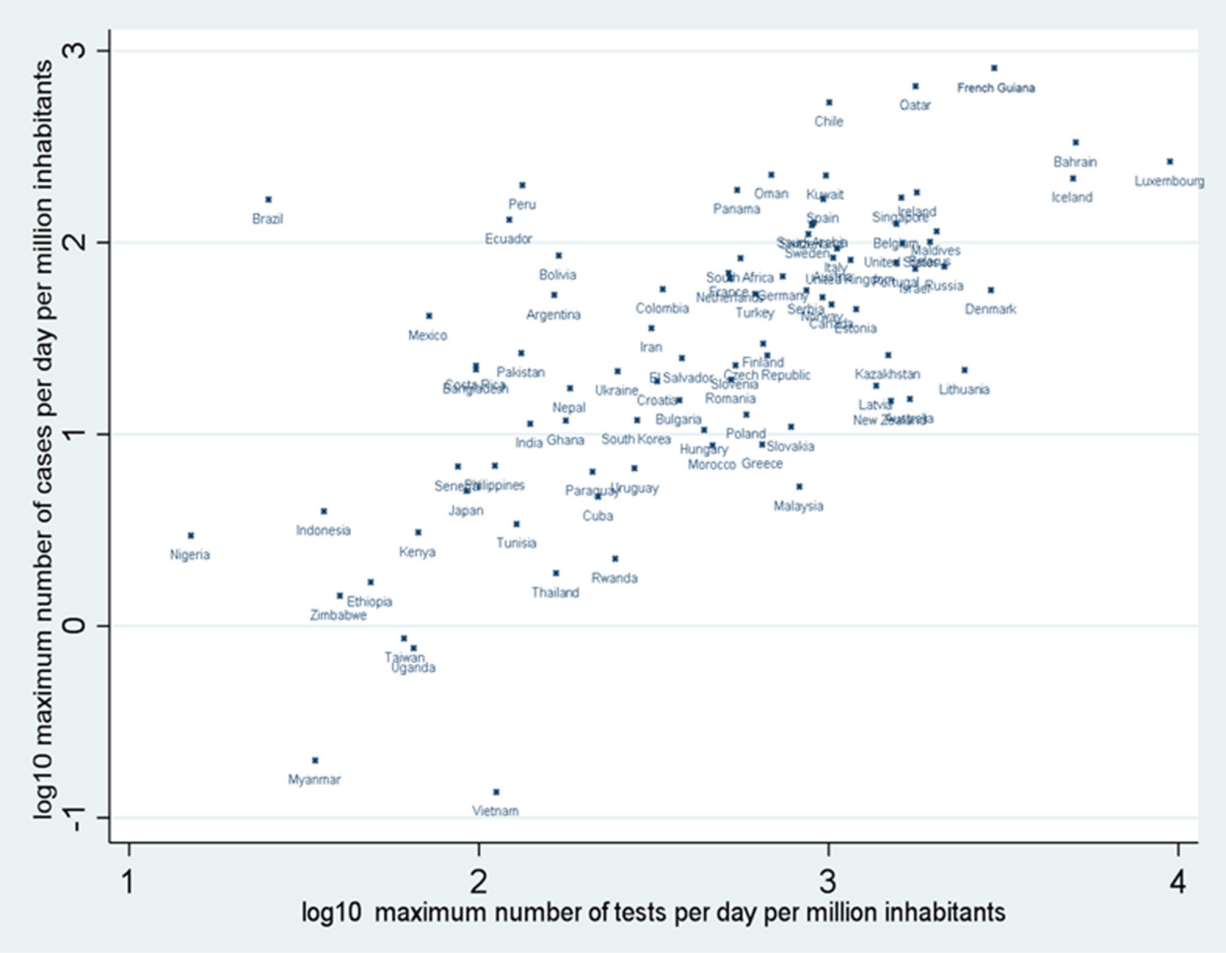
Scatter plot of the log_10_ or the maximum number of cases per day per million inhabitants and the log_10_ of the number of persons tested per day millions inhabitants in selected countries and Amazonian territories.

Suriname and Guyana had relatively few cases and the cumulated number of tests was low; French Guiana had a late epidemic surge (passed the 100th case threshold on April 24th) with intense testing scale-up in May and June 2020 often exceeding 4,000 persons tested daily per million inhabitants, levels only surpassed by Bahrain, Iceland, and Luxembourg (Figure 5). Figure 5 also shows that for countries with Amazonian territories with available daily testing data (Brazil, Peru, Bolivia, Colombia, and Ecuador) the maximum daily number of tests at the whole country level was low compared to the maximum number of cases, notably Brazil which was an outlier.

### Median Daily R_t_ Values Over Time

**Figure 6:**
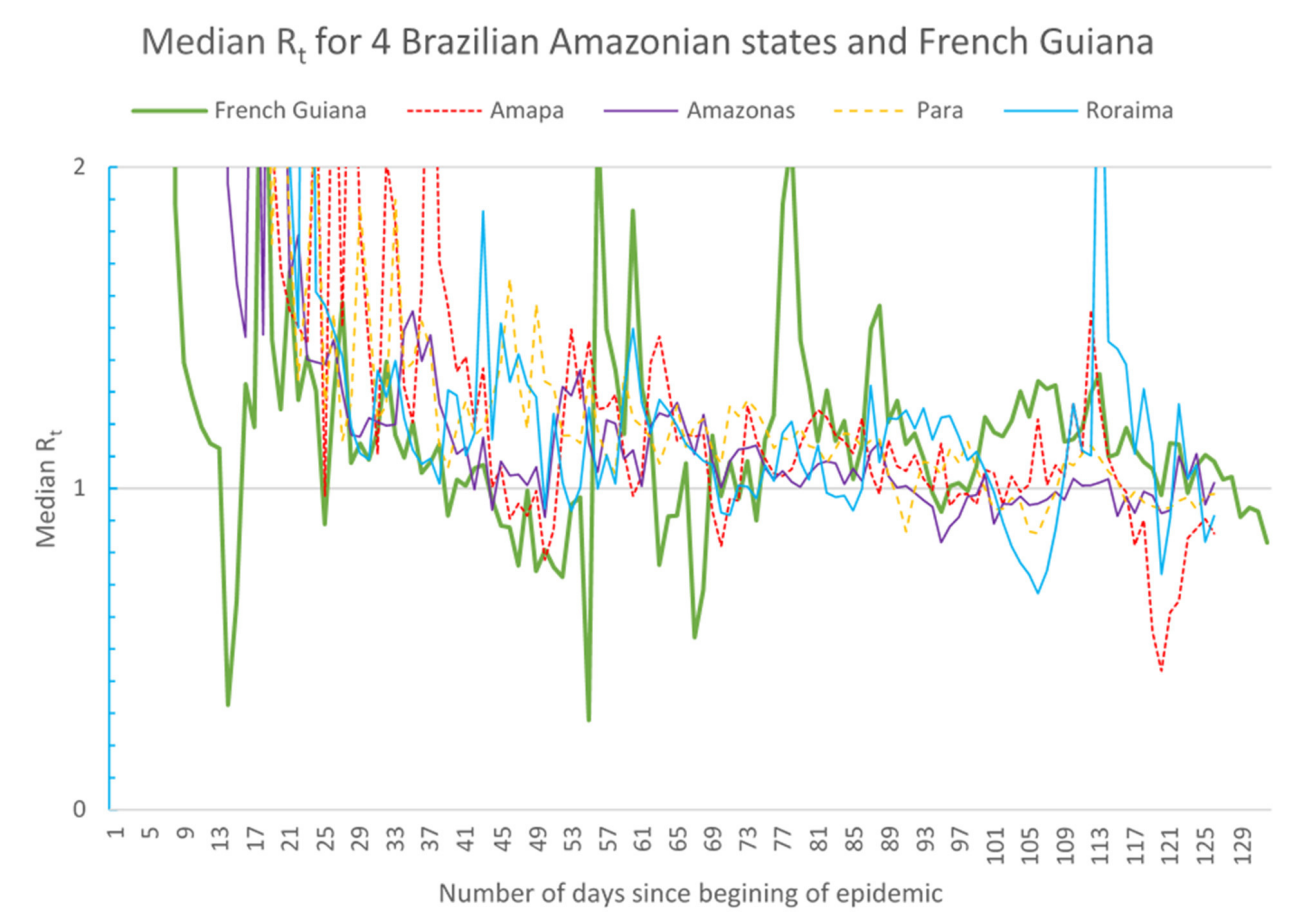
Evolution of the median Rt in Amazon territories.

Figure 6 shows the median daily R_t_ values for French Guiana, Amapá, Amazonas, Pará, and Roraima. Generally, apart from large daily fluctuation, the median values were between 1 and 1.5 and in their latest estimation were below 1.

## Discussion

The present results show that the spread of the COVID 19 epidemic in the Amazon was slower that in Europe, North America or Southern Brazil. Despite daily fluctuations median R_t_ values for territories with available data were generally slightly above 1 falling recently below 1. Amapa, the neighboring state with French Guiana seemed to be leading the trends observed in French Guiana. Perhaps lower population densities, scarce transport infrastructure, climatic factors, and perhaps the benefit of the knowledge of what had just happened in Asia and Europe slowed transmission. Nevertheless, transmission did occur and the curves seemed to take a longer time to “break” perhaps reflecting its gradual spread from community to community along the ramifications of social networks, often by boat (27–29). The heterogeneity between Amazonian territories is also a remarkable feature that reflects the scarce transport infrastructure, the very early lockdown of borders in early March, and obvious political commitment levels to tackle the problem. For Guyana, Suriname, and French Guiana, territories with few hospital and ICU beds the perspective of an overwhelming surge led early on to a lock down with interruption of air traffic, border closure, social distancing, confinement, quarantine, and isolation of patients. For Brazil, daily airline connection with major cities in southern Brazil presumably rapidly fueled the local epidemic in the North. In addition, there has been a great confusion between Federal, State and municipal levels reflected by the resignation of 2 health ministers, and the current absence of one to this date. It is very likely that the polarized political context has had a significant impact in the very high number of cases and deaths in the Amazonian States of Brazil, and beyond. For French Guiana and Suriname, after a first “phony war” period with very few cases imported from Europe which were easily controlled, the epidemic in the Amazonian states permeated across the border and with the lift of confinement, and perhaps the belief that there would be no epidemic after all, it gradually spread mostly across the most precarious communities, reaching all ethnic groups, despite uninterrupted contact tracing, and intensive community mobilization and testing.

Regarding the number of deaths, there was great heterogeneity. Studies have suggested that high ultraviolet radiation was associated with lower COVID 19 mortality (16–18) and that vitamin D deficiency (30), a frequent feature in Latin America, was associated with greater mortality. Although these factors may marginally affect mortality, it seems the explanation for such heterogeneity lies elsewhere. First despite the young age of the populations in the North, the magnitude of the number of cases in the Northern Brazilian states may have simply overwhelmed the health system and led to suboptimal care; Although the statistical plots between confirmed cases and deaths suffers from the imprecision of not reflecting testing efforts, and testing seemed relatively low in Brazil. Presumably the real number of COVID 19 cases was much greater that the number of confirmed cases (31). However, the raw numbers of deaths and the sights of mass graves reported in much of the press demonstrate how massive the influx of severe patients was. Among of the particularities of the Northern states are the high levels of social deprivation, and its ethnic makeup which perhaps leads to greater levels of acquisition and mortality (32–39). The picture in French Guiana, however, was quite singular. There is a large poor immigrant population, many vulnerable ethnic groups, high levels of comorbidities such as diabetes, obesity, and hypertension, which were expected to increase the risk of severe forms and deaths (40). However, to this day the levels of mortality are very low. Among the potential explanations, we can cite the youth of the population (median 22 years), but it is of note that it is the same as that of Amapá, where case numbers per million were 2.1 times greater than in French Guiana and mortality per million was 7.8 times greater than in French Guiana. The intense scaling up of testing and contact tracing presumably led to a denominator that is close to the real number of cases in French Guiana. The surge of cases in French Guiana occurred between June and July and presumably care of patients benefitted from knowledge accumulated since the beginning of the epidemic: hence telemedicine for patients remaining at home and the aggressive search for any sign of silent hypoxia, anticoagulation, the use of steroids when pneumonia worsens, progress in ICU ventilation methods (such as nasal high flow therapy, prone position in intubated but also non-intubated COVID-19 patients), and early massive organizational efforts to expand hospital and ICU beds for COVID patients (41) all combined to reduce mortality in French Guiana.

The limitations of this study are that it relies of aggregated data from different information systems, with incomplete knowledge of policies, dates of implementation at different locations. Causal inferences are impossible with such data. Nevertheless, the strength of this study is that it attempts to focus on the Amazon basin and to compare between territories within the region and other countries with very different context, showing instructive contrasts.

In conclusion, despite a number of factors that were expected to slow down the spread of the virus, the epidemic spread widely in the Amazonian regions, and led to considerable mortality in Northern Brazil. Widespread poverty, low access to care in remote areas, ethnic factors, comorbidities, and political denial of the health crisis may have explained the tragic situation. By contrast French Guiana, which aggressively tackled the crisis early on observed a delayed epidemic with low mortality, showing as elsewhere that despite a number of challenges it is possible to significantly impact transmission and mortality and reduce the impact of an uncontrolled surge in severe COVID 19 infections. The present analysis also suggests that an integrated data analysis is possible at the regional level and that learning from comparing territories in this complex Amazonian context may help health authorities optimize the response to the massive crisis caused by this novel pathogen (42).

## Data Availability Statement

Publicly available datasets were analyzed in this study. This data can be found at: https://ourworldindata.org/, https://covid.saude.gov.br/.

## Author Contributions

MN analyzed data, wrote first draft, and validated final manuscript. CR, TS, and AAn collected data and edited manuscript. MG, CM, VS, MDo, RS, AAd, MDe, PA, LE, and FD data interpretation and manuscript edition.

## Conflict of Interest

The authors declare that the research was conducted in the absence of any commercial or financial relationships that could be construed as a potential conflict of interest.
