# Supplementary figures and images for: The Epidemiology of COVID 19 in the Amazon and the Guianas: Similarities, Differences, and International Comparisons
Source: Front Public Health. 2021 Mar 11;9:586299. doi: 10.3389/fpubh.2021.586299 (PMC7990879; doi:10.3389/fpubh.2021.586299)

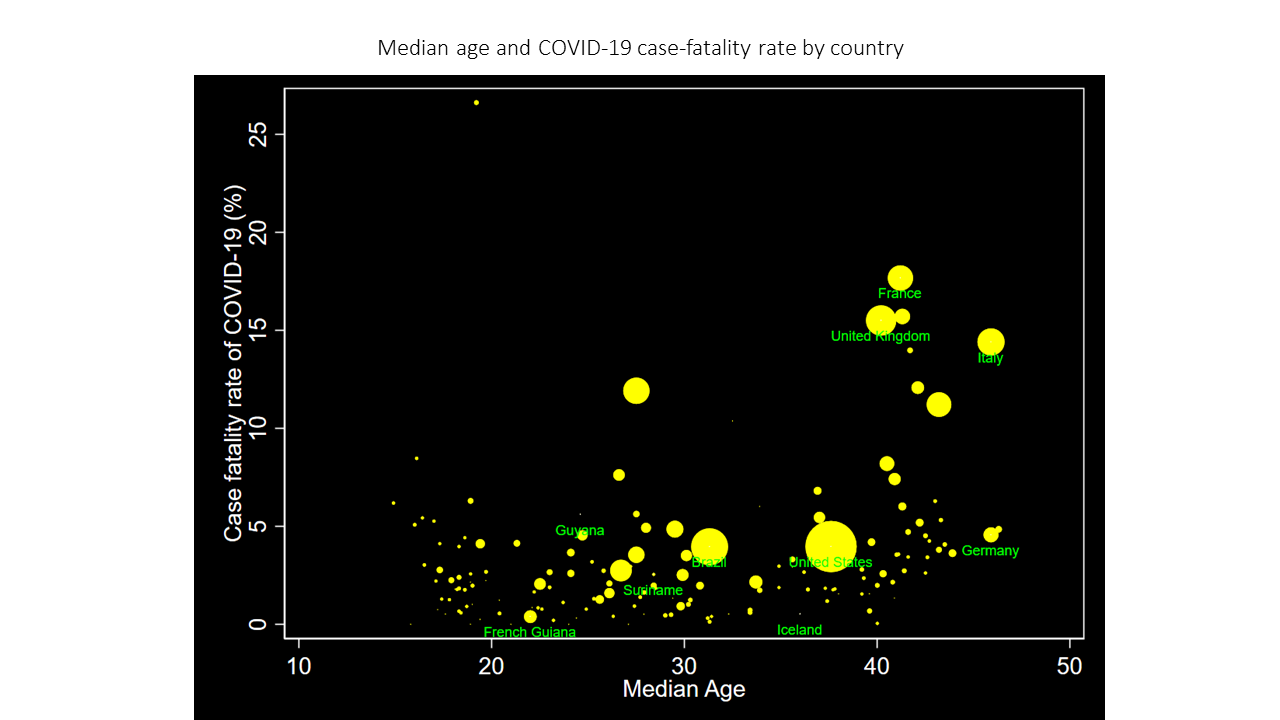

Supplement: Supplementary file 1 [file Image_1.TIF]
